# Survival from breast cancer in women with a BRCA2 mutation by treatment

**DOI:** 10.1038/s41416-020-01164-1

**Published:** 2021-02-18

**Authors:** D. Gareth Evans, Kelly-Anne Phillips, Roger L. Milne, Robert Fruscio, Cezary Cybulski, Jacek Gronwald, Jan Lubinski, Tomasz Huzarski, Zerin Hyder, Claire Forde, Kelly Metcalfe, Leigha Senter, Jeffrey Weitzel, Nadine Tung, Dana Zakalik, Maria Ekholm, Ping Sun, Steven A. Narod, kConFab Investigators, Polish Hereditary Breast Cancer Consortium, Hereditary Breast Cancer Clinical Study Group, Maria Błasińska-Morawiec, Maria Chosia, Kazimierz Drosik, Sylwia Gozdecka-Grodecka, Stanisław Goźdź, Ewa Grzybowska, Arkadiusz Jeziorski, Aldona Karczewska, Radzisław Kordek, Agnieszka Synowiec, Beata Kozak-Klonowska, Katarzyna Lamperska, Dariusz Lange, Andrzej Mackiewicz, Jerzy Władysław Mituś, Stanislas Niepsuj, Oleg Oszurek, Karol Gugała, Zbigniew Morawiec, Tomasz Mierzwa, Michał Posmyk, Janusz Ryś, Cezary Szczylik, Michał Uciński, Krzysztof Urbański, Bernard Waśko, Piotr Wandzel, Michael Friedlander, Sue Anne McLachlan, Stephanie Nesci, Sandra Picken, Sarah O’Connor, Lucy Stanhope, Andrea Eisen, Kevin Sweet, Raymond Kim, William Foulkes, Pal Moller, Susan Neuhausen, Carey Cullinane, Charis Eng, Peter Ainsworth, Fergus Couch, Christian Singer, Beth Karlan, Wendy McKinnon, Marie Wood

**Affiliations:** 1 Genomic Medicine, Manchester Academic Health Science Centre, The University of Manchester, Manchester, UK; 2 Sir Peter MacCallum Department of Oncology, University of Melbourne, Parkville, Australia; 3 Department of Medical Oncology, Peter MacCallum Cancer Centre, Melbourne, Australia; 4 Centre for Epidemiology and Biostatistics, Melbourne School of Population and Global Health, The University of Melbourne, Melbourne, VIC Australia; 5 Precision Medicine, School of Clinical Sciences at Monash Health, Monash University, Clayton, VIC Australia; 6 Cancer Epidemiology Division, Cancer Council Victoria, Melbourne, VIC Australia; 7 Department of Medicine and Surgery, University of Milan-Bicocca, Milan, Italy; 8 Department of Genetics and Pathology, International Hereditary Cancer Center, Pomeranian Medical University, Szczecin, Poland; 9 University of Zielona Góra, Zielona Góra, Poland; 10 Women’s College Research Institute, Women’s College Hospital, Toronto, ON Canada; 11 Division of Human Genetics, the Ohio State University Medical Center, Comprehensive Cancer Center, Columbus, OH USA; 12 Division of Clinical Cancer Genomics, Department of Population Sciences, City of Hope, Duarte, CA USA; 13 Beth Israel Deaconess Medical Center, Boston, MA USA; 14 Nancy and James Grosfeld Cancer Genetics Center, Beaumont Cancer Institute, Beaumont Health, Royal Oak, MI USA; 15 Manchester Academic Health Science Centre, Department of Medical Oncology, The Christie, Manchester, UK; 16 Copernicus Memorial Hospital, Lodz, Poland; 17 Pomeranian Medical University, Szczecin, Poland; 18 Regional Cancer Center, Opole, Poland; 19 Poznan University of Medical Sciences, Poznań, Poland; 20 Holycross Cancer Centre, Kielce, Poland; 21 Maria Skłodowska-Curie Institute, Gliwice, Poland; 22 Medical University of Lodz, Lodz, Poland; 23 University School of Medical Sciences at Great Poland Cancer Center, Poznań, Poland; 24 Military Institute of Medicine, Warsaw, Poland; 25 Holycross Cancer Centre, Kielce, Poland; 26 Greater Poland Cancer Centre, Poznan, Poland; 27 Maria Sklodowska-Curie Institute-Oncology Center, Gliwice, Poland; 28 Maria Sklodowska-Curie Memorial Cancer Centre and Institute of Oncology, Krakow, Poland; 29 M. Skłodowska-Curie Memorial Institute of Oncology, Krakow, Poland; 30 Regional Oncology Hospital, Olsztyn, Poland; 31 Regional Oncology Hospital, Bydgoszcz, Poland; 32 Regional Oncology Center, Białystok, Poland; 33 Maria Sklodowska-Curie Memorial Cancer Center and Institute of Oncology, Krakow, Poland; 34 Military Hospital, Warsaw, Poland; 35 Institute of Oncology, Krakow, Poland; 36 Regional Hospital, Rzeszów, Poland; 37 Regional Hospital Bielsko-Biała, Bielsko-Biała, Poland; 38 Prince of Wales Hospital, Randwick, Australia; 39 St Vincent’s Hospital, Fitzroy, Australia; 40 Peter MacCallum Cancer Centre, Melbourne, Australia; 41 Sunnybrook Health Sciences Centre, Toronto, Canada; 42 Ohio State University, Columbus, Ohio USA; 43 University Health Network, Toronto, Canada; 44 McGill University, Montreal, Canada; 45 Norwegian Radium Hospital, Oslo, Norway; 46 City of Hope, Duarte, CA USA; 47 City of Hope National Medical Center, Duarte, CA USA; 48 Cleveland Clinic, Cleveland, Ohio USA; 49 London Health Sciences Centre, London, Canada; 50 Mayo Clinic, Rochester, MN USA; 51 Medical University of Vienna, Vienna, Austria; 52 University of California, Los Angeles, CA USA; 53 University of Vermont, Burlington, VT USA

**Keywords:** Targeted therapies, Breast cancer

## Abstract

**Background:**

The impact of various breast-cancer treatments on patients with a BRCA2 mutation has not been studied. We sought to estimate the impact of bilateral oophorectomy and other treatments on breast cancer-specific survival among patients with a germline *BRCA2* mutation.

**Methods:**

We identified 664 women with stage I–III breast cancer and a BRCA2 mutation by combining five different datasets (retrospective and prospective). Subjects were followed for 7.2 years from diagnosis to death from breast cancer. Tumour characteristics and cancer treatments were patient-reported and derived from medical records. Predictors of survival were determined using Cox proportional hazard models, adjusted for other treatments and for prognostic features.

**Results:**

The 10-year breast-cancer survival for ER-positive patients was 78.9% and for ER-negative patients was 82.3% (adjusted HR = 1.23 (95% CI, 0.62–2.45, *p* = 0.55)). The 10-year breast-cancer survival for women who had a bilateral oophorectomy was 89.1% and for women who did not have an oophorectomy was 59.0% (adjusted HR = 0.45; 95% CI, 0.28–0.72, *p* = 0.001). The adjusted hazard ratio for chemotherapy was 0.83 (95% CI, 0.65–1.53: *p* = 0.56).

**Conclusions:**

For women with breast cancer and a germline *BRCA2* mutation, positive ER status does not predict superior survival. Oophorectomy is associated with a reduced risk of death from breast cancer and should be considered in the treatment plan.

## Background

The choice of treatment for a patient with early breast cancer takes into account tumour features, including tumour size, grade, hormone-receptor status, HER2 status, axillary nodal status and patient preferences. Evidence is emerging that knowledge of inherited mutations in predisposing genes, such as BRCA1 and BRCA2, is also important.^1–4^ The majority of *BRCA2*-associated breast cancers are ER-positive, and in the general population, ER positivity is a favourable prognostic factor, compared to ER negativity early in the course of the disease.^5^ However, recent studies indicate that positive ER status and low tumour grade may not predict a good outcome in *BRCA2* carriers.^6,7^

A beneficial effect of oophorectomy on breast-cancer survival has been seen in *BRCA1* mutation carriers^1–3^ with hazard ratios ranging from 0.4 to 0.6. It is important to confirm that oophorectomy is helpful in the treatment of breast cancer in patients with a *BRCA2* mutation. Many of the patients are premenopausal, and oophorectomy will curtail fertility and induce surgical menopause, which increases risks for osteoporosis, cardiovascular disease and possibly cognitive dysfunction.^8^ In general, hormone-replacement therapy is not advised for women with ER-positive breast cancer. It is not likely that a randomised clinical trial will be conducted in *BRCA2* mutation carriers; therefore, we must rely on large, well-conducted observational studies. Our primary goal was to assess the efficacy of various treatments in reducing breast-cancer mortality in the *BRCA2* patient population. We combined data from five different databases and conducted a multicentre historical cohort study of women with breast cancer and a *BRCA2* mutation, resident in North America, Europe and Australia, to identify the impact of various treatments (chemotherapy, oophorectomy, hormonal therapy, contralateral mastectomy and radiotherapy) on survival.

## Methods

### Study subjects

Patients were eligible for the study if they had a diagnosis of stage I–III invasive breast cancer that was pathologically confirmed and carried a germline *BRCA2* mutation. We excluded patients with known metastatic disease at diagnosis. Age at diagnosis was restricted to 25 and 70 years and year of diagnosis was between 1990 and 2019. Patients were eligible if they had genetic testing within 5 years of the diagnosis of breast cancer (2 years in Australia) and were found to be *BRCA2*-positive. Patients were also eligible if they developed breast cancer after genetic testing (i.e., in the follow-up period). Patients with a previous diagnosis of cancer in the same or contralateral breast or cancer at another site were excluded. We wished to study the impact of oophorectomy after diagnosis, and therefore patients who had a bilateral oophorectomy prior to their breast-cancer diagnosis were excluded. The indication for oophorectomy was not recorded.

### Patient sources

#### North America

Eligible study subjects included female *BRCA2* mutation carriers from 67 participating centres in Canada and the United States who were enrolled in a multicentre, longitudinal cohort study. All subjects sought testing for *BRCA1* and *BRCA2* mutations because of a personal or family history of breast and/or ovarian cancer. Mutation detection was performed using a range of techniques, but all nucleotide sequences were confirmed by direct sequencing of DNA. The study was approved by the institutional ethics review boards of the respective host institutions and all study subjects provided written informed consent. All subjects completed a baseline research questionnaire at the time of a clinic appointment or at their home at a later date. Subjects were eligible if they were cancer-free at the time of study enrolments (the baseline questionnaire) and reported a new diagnosis of invasive breast cancer in one of the follow-up questionnaires. They were also eligible if they had been diagnosed with breast cancer in the 5-year period prior to genetic testing. The questionnaires requested detailed information on family and personal history of cancer, reproductive and medical histories as well as medication use. Follow-up questionnaires are completed every 2 years thereafter to update exposure information and to capture incident disease and deaths from all causes. Information on diagnoses and treatment of breast cancer was collected from the questionnaires and from review of medical records. Hormone-receptor status of the tumour and other pathologic features were abstracted from the pathology report and/or medical record review. Cause and date of death was obtained by the collaborating investigator at each of the participating sites. This was determined by patient record, by correspondence with the treating physician or by next of kin.

#### Poland

A *BRCA2* mutation is present in approximately 2% of the familial breast-cancer cases in Poland.^9^ Since 1996, the team has collected blood samples for DNA extraction from unselected women with newly diagnosed breast cancer in various clinical centres across Poland. From 1996 to 2001, this was done primarily for young breast-cancer patients (age of diagnosis <50), and from 2002 onwards, included breast cancer patients diagnosed at all ages. In total, DNA samples from approximately 13 000 breast-cancer patients were collected between 1996 and 2014. The patients were tested for the presence of two founder mutations in *BRCA2*. The patients in the study were those who were found to be positive for one of the two mutations. The mean time from diagnosis to blood testing was 1.4 years. Patients had no prior diagnosis of breast cancer or another cancer. Information on clinical presentation and treatments received was retrieved from the medical records. Vital status and date of death was retrieved by linkage to the Vital Statistics Database of the Polish Ministry of Administration and Internal Affairs. Cause of death was determined by medical record review and interview with the treating physician.

#### Australia

Between 1997 and 2008, more than 6000 women enrolled in the Kathleen Cuningham Foundation Consortium for Research into Familial Breast Cancer (kConFab).^10^ They have been systematically followed up with a mailed questionnaire at 3-yearly intervals as part of the kConFab Follow-Up Study.^11^ Study participants are predominantly from families with multiple cases of breast and/or ovarian cancer and were recruited from one of 16 Familial Cancer Centres across Australia and New Zealand. At the time of enrolment, blood was collected for *BRCA1* and *BRCA2* mutation analysis. The baseline and 3-yearly follow-up questionnaires ask about demographics, cancer risk factors, cancer diagnoses, surgeries (treatment and prophylactic) and cancer treatments.^12^ Breast cancers are verified by obtaining pathology reports and medical records. Vital status and cause of death was obtained by relative report or from the death registry. The kConFab Follow-up Study includes 508 women with a *BRCA2* germline mutation with a mean of 11.3 years of follow-up; 278 of these were affected with Stage I–III invasive breast cancer either before baseline or during follow-up. Patients were included if there was no prior history of cancer, if they were between 25 and 70 years of age and if less than 2 years had elapsed between the date of breast cancer and the date of genetic testing.

#### United Kingdom

The source of patients was a clinical database of *BRCA1* and *BRCA2* mutation carriers initiated in Manchester in 1999. This includes all carriers of pathogenic mutations in *BRCA2* identified through genetic testing in the Manchester region. The regional testing laboratory opened in 1996. Data include all treatment details relating to breast and ovarian cancer and the associated pathology. Vital status is determined through NHS systems.

#### Italy

The source of patients was the clinical database of *BRCA2* mutation carriers treated at the outpatient clinic of the San Gerardo hospital in Milan between 2007 and 2019. Data were retrieved for women who had identified a mutation prior to 2018. All patients participated in an ongoing cohort study and all indicated their willingness to be followed. Each of the 57 patients enrolled in the study was contacted via email or by phone, and details about the pathological examination of breast cancer were recorded. Details about treatment received were collected from the patients and from review of clinic notes and hospital records.

The nature of patient selection and the testing process varied by centre (see above), but all patients carried a confirmed pathogenic mutation in *BRCA2*. For each case, the medical record was reviewed, and information on age of diagnosis, tumour size (cm) and nodal status (positive/negative) was obtained. Information was also obtained from the questionnaires (patient- reported outcomes). Grade, oestrogen-receptor status (positive/negative), progesterone-receptor status (positive/negative) and HER2 status (positive/negative) was recorded as assigned by the pathologist associated with the hospital of treatment. Chemotherapy, tamoxifen, aromatase inhibitors and radiotherapy were recorded as yes/no. Most studies did not routinely record the specific chemotherapy formulation or the timing of administration (neoadjuvant or adjuvant) or use of ovarian-suppression therapy. Oophorectomy was recorded (yes/no) as well as the date of oophorectomy. In general, most operations are bilateral salpingo-oophorectomy (TAH-BSO). Hysterectomy is done in some centres but not all. Oophorectomies that were done for the treatment of ovarian cancer, metastatic breast cancer or other cancer were considered as no oophorectomy. We recorded the date of contralateral mastectomy; some were done as initial surgical treatment and some were done at a later date. We recorded the dates of all contralateral breast cancers.

### Statistical analysis

Survival analysis was conducted using Cox proportional hazard models; subjects were followed from the date of diagnosis (or date of genetic testing, whichever came last) until age 80, the date of completion of the last questionnaire or date last known alive, ovarian cancer, pancreatic cancer, death from breast cancer or death from another cause. Women for whom the cause of death was missing were censored as death from another cause. To adjust for potential survivorship bias that might result because genetic testing was done after diagnosis, follow-up was left-truncated at the date of genetic testing. We estimated the hazard ratio for breast-cancer-specific death associated with radiotherapy (yes/no), chemotherapy (yes/no), tamoxifen/AI use and oophorectomy. Oophorectomy and contralateral mastectomy were treated as time-dependent covariates. The covariates included tumour size (0–1.9 cm, 2.0–4.9 cm and 5.0 cm+), nodal status (negative/positive), ER status (+/−), PR status (+/−/missing), HER2 (+/−/missing) and tumour grade (I/II vs. III). The analysis was repeated restricting the study set to ER-positive breast-cancer patients. The analysis was also repeated for subgroups according to age at diagnosis (<40; 40–50 ≥ 50 years).

A secondary matched analysis was done specifically to study the effect of oophorectomy on survival. In this study, each woman who had an oophorectomy was matched to a woman who had not. Pairs were generated, matched on date of birth (within 4 years), year of genetic test (within 2 years) and country. Further, to be matched to an exposed woman, the unexposed women had to have an age of the last follow-up older than age at the date of oophorectomy in the matched exposed woman. The unexposed control had an age at diagnosis younger than that of the exposed woman. Both subjects were followed for death from the date of oophorectomy in the case. The date of oophorectomy in the exposed woman was used as the date of the initial follow-up for the control. The hazard ratio generated for the matched pairs was adjusted for tumour size, nodal status and ER status using a Cox proportional hazard model.

We also assessed time from diagnosis to oophorectomy as a predictor of mortality. The average time elapsed from diagnosis to oophorectomy was 3.2 years. In total, 82 of the oophorectomies were done within the first year following diagnosis. We assessed the hazard ratio in the Cox model for three subgroups of patients based on time from breast surgery to oophorectomy (less than 1 year, 1–3 years and 3 or more years), compared to women who never had an oophorectomy. We also conducted an analysis comparing women who had an oophorectomy in the first year with all other women (no oophorectomy and later oophorectomy).

## Results

Summary information for the 664 patients is presented in Table 1 and divided by centre in Supplementary Table 1. The mean age of diagnosis was 44.5 years (range 26–70 years); 68.5% of cancers were ER-positive and 41.9% were lymph-node-positive. The median time of follow-up was 6.3 years (range 0.1–27.9 years). Among those with ER status reported, the proportion that was ER-positive was 83% for women aged less than 40 years at diagnosis, 81% for women 40–50, 79% for women 50–60 and 76% for women of age 60 years and above. Among the ER-positive cancers, 49.9% were node-positive, and among the ER-negative cancers, 37.8% were node-positive (p = 0.03 for difference).

**Table 1:** Characteristics of the study subjects.

Variables	All subjects *N* = 664
Year of birth (range)	1961 (1933–1989)
Age at diagnosis (years, range)	44.5 (26.0–70.0)
Year of diagnosis	2006 (1990–2019)
Age at gene testing	45.3 (26.4–73.5)
Before diagnosis of breast cancer	130 (19.6%)
0–1 year after diagnosis	242 (36.5%)
1–5 years after diagnosis	292 (44.0%)
5+ years after diagnosis	0
Mean time elapsed (years) from diagnosis to genetic testing	1.4 (0–5.0)
Age at the last follow-up (years, range)	52.1 (28.9–80.0)
Years of follow-up	7.6 (0.1–27.9)
Contralateral breast cancer	29 (5.0)
Ovarian cancer	11 (1.7)
Death	
No	552 (83.1%)
Yes	112 (16.9%)
Cause of death	
Breast cancer	91
Other	9
Unknown/missing	12
Tumour grade	
I	50 (7.5%)
II	198 (29.8%)
III	236 (35.5%)
Missing	180 (27.1%)
Tumour size	
Mean (mm)	22.6 (0–300)
<=10	127 (19.1%)
11–20	222 (33.4%)
21+	233 (35.1%)
Missing	82 (12.3%)
ER status	
Positive	455 (68.5%)
Negative	107 (16.1%)
Missing	102 (15.4%)
PR status	
Positive	274 (41.3%)
Negative	101 (15.2%)
Missing	289 (43.5%)
HER2 status	
Positive	43 (9.6%)
Negative	200 (44.8%)
Missing	421 (45.6%)
Lymph-node status	
Negative	310 (46.7%)
Positive	278 (41.9%)
Missing	76 (11.5%)
Endocrine therapy	
Neither	198 (29.8%)
Tamoxifen alone	308 (46.4%)
AI alone	49 (7.4%)
Both	23 (3.5%)
Missing	86 (13.0%)
Chemotherapy	
No	165 (24.9%)
Yes	419 (63.1%)
Missing	80 (12.1%)
Contralateral mastectomy	
No	345 (52.0%)
Yes	307 (46.2%)
Missing	12 (1.8%)
Oophorectomy	
No	287 (43.2%)
Yes	377 (56.8%)
Oophorectomy within 1 year of breast cancer	82 (21.7%)
Oophorectomy after 1 year	295 (78.3%)
Mean years from breast cancer to oophorectomy	3.2 (1–17.0) *n* = 295

The majority of the patients with ER-positive breast cancer received endocrine therapy: tamoxifen alone (269, 66.4%) an AI alone (43, 10.6%) or both (18, 4.4%). When women with missing data were excluded, 419 (71.8%) of all patients received chemotherapy (neoadjuvant or adjuvant) and 248 (67.0%) received both endocrine therapy and chemotherapy. About 46.2% had a contralateral mastectomy and 56.8% had a bilateral oophorectomy after diagnosis. The proportion of women who had an oophorectomy was similar for women with ER-positive (58.7%) and ER-negative breast cancer (55.1%).

**Fig. 1:**
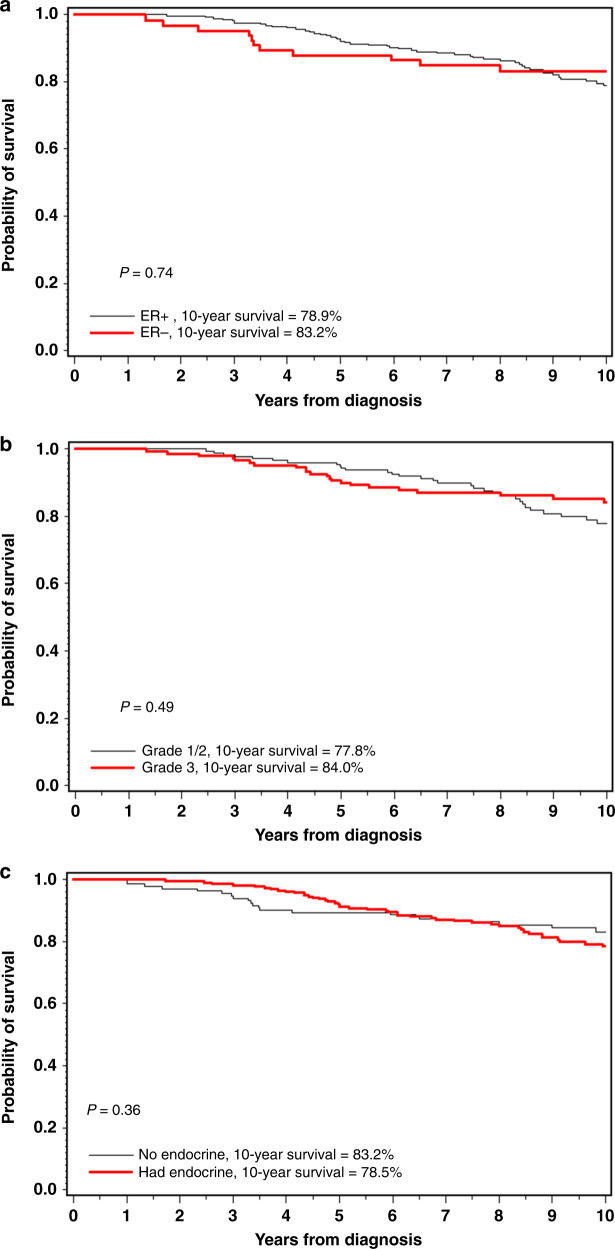
Ten-year breast cancer-specific survival for women with BRCA2 mutations. **a** ER-positive versus ER-negative. **b** Grade 1/2 versus Grade 3. **c** Endocrine therapy versus no endocrine therapy.

Overall, there were 112 deaths in the cohort; of these, 91 were from breast cancer and nine were from causes other than breast cancer (Supplementary Table 2). For 12 women, the cause of death was missing. Both tumour size and axillary nodal status were prognostic, but ER status and tumour grade were not (Supplementary Table 3 and Table 2 and Figs. 1a, b).

**Table 2:** Hazard ratios for death from breast cancer for selected variables.

Variables	Cases/total	Univariate HR (95% CI) *P* value	Multivariate^a^ HR (95% CI) *P* value
Age at diagnosis			
<=40	38/243	1	1
41–50	31/252	0.81 (0.51–1.31) 0.39	0.94 (0.58–1.53) 0.81
50+	22/169	1.00 (0.60–1.79) 0.97	1.01 (0.58–1.75) 0.98
BC size			
<=10	7/127	1	1
11–20	31/222	2.95 (1.30–6.69) 0.01	2.41 (1.04–5.58) 0.04
>20	39/233	4.09 (1.83–19.2) 0.0006	3.13 (1.33–7.35) 0.009
Endocrine therapy			
None	24/198	1	1
Tamoxifen alone	45/308	1.36 (0.83–2.23) 0.23	1.11 (0.62–1.98) 0.73
AI alone	6/49	1.45 (0.59–3.55) 0.42	1.56 (0.56–4.33) 0.40
Both	3/23	0.98 (0.30–3.26) 0.98	1.24 (0.35–4.42) 0.74
Either	54/380	1.34 (0.84–2.17) 0.23	1.13 (0.64–2.00) 0.67
Grade			
1/2	34/248	0.97 (0.59–1.58) 0.89	1.17 (0.70–1.96) 0.55
3	31/236	1	1
ER status			
Negative	12/107	1	1
Positive	60/455	1.29 (0.69–2.39) 0.43	1.23 (0.62–2.45) 0.55
Nodal status			
Negative	34/310	1	1
Positive	48/278	1.83 (1.18–2.84) 0.007	1.67 (1.00–2.79) 0.05
Chemotherapy			
No	19/165	1	1
Yes	54/419	1.24 (0.73–2.09) 0.42	0.83 (0.65–1.53) 0.56
Contralateral mastectomy			
No	58/345	1	1
Yes	32/307	0.56 (0.36–0.88) 0.009	0.71 (0.44–1.15) 0.17
Missing	1/12		
Oophorectomy			
No	59/287	1	1
Yes	32/377	0.37 (0.24–0.58) <0.001	0.45 (0.28–0.72) 0.0009
Yes, within 1 year	8/82	0.45 (0.21–0.93) 0.03	0.56 (0.26–1.21) 0.14
Yes, 1–2 years after diagnosis	18/184	0.42 (0.25–0.71) 0.001	0.51 (0.29–0.91) 0.02
Yes, 3+ years after diagnosis	6/111	0.23 (0.10–0.55) 0.001	0.28 (0.12–0.67) 0.004
Yes, age at ooph <50	24/245	0.42 (0.26–0.67) 0.0004	0.51 (0.30–0.87) 0.01
Y, age at ooph >=50	8/132	0.28 (0.13–0.60) 0.0009	0.33 (0.15–0.74) 0.007

^a^Adjusted by all the variables; the 12 subjects missing PM data were supposed as no PM in the estimation on the RR of factors other than PM.

### Tamoxifen/endocrine therapy

Among the 455 ER-positive patients, the 10-year survival for those who had taken any endocrine therapy was 78.8% and for those who had taken no endocrine therapy was 82.8% (adjusted HR = 1.48, 95% CI, 0.69–3.20;, p = 0.31). The adjusted hazard ratio for any endocrine therapy (tamoxifen or AI) versus no endocrine therapy was 1.48 (95% CI, 0.69–3.20, *p* = 0.31). The adjusted hazard ratio for tamoxifen (vs. no endocrine therapy) was 1.57 (95% CI, 0.72–3.41, *p* = 0.25). The adjusted hazard ratio for AI alone versus no endocrine therapy was 1.23 (95% CI, 0.30–5.04, *p* = 0.77). Figure 1c illustrates the crude survival of the women who did and did not receive endocrine therapy.

### Chemotherapy

Among all 664 patients, the adjusted hazard ratio for chemotherapy (yes/no) was 0.83 (95% CI, 0.65–1.53, *p* = 0.56). Among the 455 ER-positive women, the adjusted hazard ratio for chemotherapy was 1.05 (95% CI, 0.49–2.24, *p* = 0.90), and among the 107 ER-negative women, the hazard ratio for chemotherapy was 0.48 (95% CI, 0.08–3.01, *p* = 0.43).

### Contralateral mastectomy

About 307 of the patients had a contralateral mastectomy; the prevalence ranged from 16.1% in Poland to 67.1% in Australia. The mean interval from initial to contralateral surgery was 2.0 years (range 0–20 years). The adjusted hazard ratio for breast-cancer death associated with contralateral mastectomy (time-dependent) was 0.71 (95% CI, 0.44–1.15, *p* = 0.17).

### Radiotherapy

Among the 664 women, the adjusted hazard ratio for radiotherapy (yes/no) on breast-cancer mortality was 0.81 (95% CI, 0.41–1.59, *p* = 0.54).

### Oophorectomy

About 377 of the patients had an oophorectomy. For 11 subjects, an oophorectomy was performed for the treatment of ovarian cancer and these were considered non-oophorectomies for purposes of the analysis (patients were censored for ovarian cancer). About 82 oophorectomies were done within a year of diagnosis and 295 were done within 1 or more years after diagnosis.

**Fig. 2:**
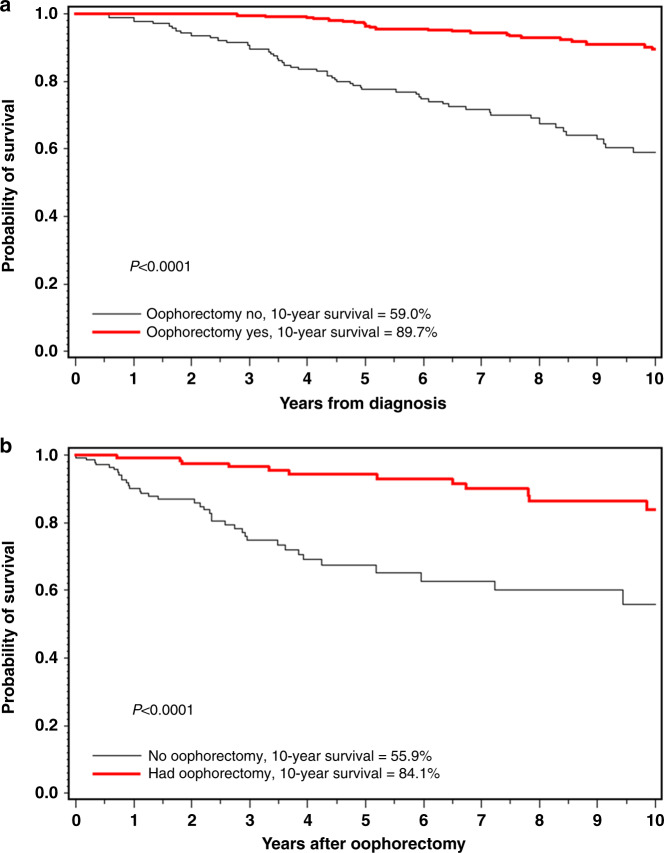
Ten-year breast cancer-specific survival for women with BRCA2 mutations. **a** Oophorectomy versus no oophorectomy (all subjects). **b** Oophorectomy versus no oophorectomy (subjects in matched analysis only).

The overall 10-year survival rate was 78.6% for all 625 women in the cohort. The 10-year survival rate was 89.7% for women who had an oophorectomy and was 59.0% for those who did not have an oophorectomy (Fig. 2a). The 10-year survival rate was 87.5% for 82 women who had an oophorectomy in the first year post diagnosis.

Among all patients, the adjusted hazard ratio for oophorectomy (time-dependent) was 0.45 (95% CI, 0.28– 0.72, *p* = 0.0009) for breast-cancer-specific death and was 0.44 (95% CI, 0.29–0.67, *p* = 0.0001) for all-cause death.

Among women under age 50 at diagnosis, the adjusted hazard ratio for oophorectomy and breast-cancer-specific death was 0.51 (95% CI, 0.30–0.87, *p* = 0.01) and for women over 50 at diagnosis it was 0.33 (95% CI, 0.15–0.74, *p* = 0.01).

The adjusted hazard ratio for oophorectomy was 0.28 (95% CI, 0.06–1.41, *p* = 0.13) for ER-negative patients and was 0.48 (95% CI, 0.25–0.86, *p* = 0.01) for ER-positive patients.

The adjusted hazard ratio for oophorectomy was 0.32 (95% CI, 0.12–0.80, *p* = 0.02) for women with small node-negative breast cancers (<2 cm) and was 0.48 (95% CI, 0.19–1.22, *p* = 0.12) for women with breast cancer that had node-positive breast cancer or greater than 2 cm.

The adjusted hazard ratio for oophorectomy was 0.55 (95% CI, 0.30–1.00, *p* = 0.05) for those who had chemotherapy and was 0.25 (95% CI, 0.07–0.88, *p* = 0.0.03) for those who did not have chemotherapy.

The adjusted hazard ratio for those who had an oophorectomy in the first year after diagnosis was 0.56 (95% CI, 0.26–1.21, *p* = 0.14) compared to those who never had an oophorectomy.

The adjusted hazard ratio for those who had an oophorectomy in the first year after diagnosis was 0.77 (95% CI, 0.37–1.63, *p* = 0.49) compared to all others (those who never had an oophorectomy and those who had an oophorectomy at a later time).

We generated 149 matched pairs for the matched analysis (Supplementary Table 4). In the matched analysis, the adjusted hazard ratio associated with oophorectomy was 0.52 (95% CI, 0.27–0.99, *p* = 0.04) (Fig. 2b).

## Discussion

The goal of our study was to assess the associations of various treatments in reducing breast-cancer mortality in the *BRCA2* patient population. We observed a decrease in the proportion of cancers that were ER-positive with age of diagnosis (rather than the expected increase) and observed that ER-positive cancers were more likely to be node-positive than ER-negative cancers. These associations have also been seen in an earlier study from our group^13^ and in the Nordic *BRCA2* mutation carrier study.^14^ Moreover, positive ER status was not predictive of better survival, compared to negative ER status.

Of the four treatments studied, oophorectomy appeared to be the most effective. The adjusted hazard ratio for breast-cancer-specific death associated with oophorectomy was 0.45 (95% CI, 0.28–0.72, *p* = 0.0009). The results were similar for the analysis of the 149 matched pairs (HR 0.52, 95% CI, 0.27–0.99). Surprisingly, the association with oophorectomy was present in women with ER-negative breast cancer, but there were only 107 ER-negative patients and the association did not reach statistical significance. It is important that possible sources of bias be considered. Oophorectomy might occur years after the diagnosis; therefore, it is important that we corrected for immortal time bias by including oophorectomy in survival models as a time-dependent covariate. We also adjusted for potential confounders, including tumour size, nodal status and contralateral mastectomy. We excluded women who had known metastatic disease at diagnosis. It is also possible that some women developed distant recurrence early in the follow-up period and that these women did not have an oophorectomy for this reason. This could bias the result towards longer survival for the oophorectomy group. However, among the women with stage I breast cancer, the hazard ratio associated with oophorectomy was 0.32 (95% CI, 0.12–0.80) and it is unlikely for these women that the choice of oophorectomy or not was based on prognosis.

Unfortunately, we did not have the dates of distant recurrence for the women in this cohort, and we cannot rule out the possibility of residual bias because of early recurrence. For this reason, we conducted a separate analysis for oophorectomy done in the first year post diagnosis. This analysis is that which closely emulates a randomised trial, where the intervention (oophorectomy) would be done in the first year of treatment. In this subgroup, the association was still significant but when we compared women with an oophorectomy in the first year with all other women (the most conservative analysis), the association was attenuated. Further, to ensure that women with and without an oophorectomy were as similar as possible, we confirmed our results in a separate matched analysis, matched on date of birth (within 4 years), year of genetic test (within 2 years) and country of residence. A protective association of oophorectomy on breast-cancer mortality was also seen in our earlier studies of BRCA1-associated breast cancer.^1–3^ It is important that these observations be confirmed in other large studies with robust methods.

There are other limitations to the study. Missing values for several prognostic factors might limit our ability to adjust for potential confounding. We had limited data on HER2 status although HER2 positivity is rare among *BRCA2* carriers.^15^ We did not distinguish between neoadjuvant and adjuvant chemotherapy, and we did not have the dates of distant recurrences. In cases where neoadjuvant chemotherapy was given, the size and nodal status was based on clinical parameters and pre-treatment imaging. Genetic testing was done for some women after diagnosis, and therefore we did a left-censored survival analysis from the date of genetic testing.

The effect of chemotherapy in *BRCA2* mutation carriers was similar to that observed in studies of non-carriers, and there is no indication from the data presented here that the decision to prescribe chemotherapy should depend on *BRCA2* carrier status.

We saw little evidence for the benefit of tamoxifen or an aromatase inhibitor in ER-positive patients in the current study (adjusted hazard ratio: any vs. neither 1.48, 95% CI, 0.69–3.20, *p* = 0.31). This could be because those treated with endocrine therapy had more aggressive cancers than those who did not get treated, but the association was adjusted for age and conventional prognostic factors. A similar lack of the effect of tamoxifen was seen in an earlier study by Goodwin et al.^16^ It is not clear if tamoxifen or aromatase inhibitors are effective treatments for ER-positive breast cancer in *BRCA2* mutation carriers. The mean follow-up in this cohort was 7.2 years and this may be insufficient to characterise the effect of a given treatment in ER-positive breast cancers. Nevertheless, it is surprising that oophorectomy appeared to be effective in this patient cohort, which did not show a beneficial effect of anti-hormonal therapy.

Increasingly, premenopausal women with breast cancer are offered ovarian suppression with a gonadotropin-releasing hormone agonist, particularly if they wish to maintain fertility, and regardless of their mutation status. We did not evaluate the effect of medical ovarian suppression on breast cancer survival in the *BRCA2* carrier population to see if medical ovarian suppression is a viable alternative to oophorectomy. In the SOFT trial,^17^ ovarian function suppression given for 5 years in addition to tamoxifen improved 8-year disease-free survival from 78.9% to 83.2% (*p* = 0.009), but this approach has not been studied in mutation carriers.

The majority of patients in the study were stage II or III (85%) and few were aware of their mutation status at the time of diagnosis. It is hoped that as genetic testing becomes more widespread, a greater proportion of patients will be diagnosed in MRI surveillance programmes and this will result in more stage I breast cancers. It will be important to revisit predictors of survival for women with MRI-detected breast cancer and for women with stage I cancers in both *BRCA2* and BRCA1 carriers.

In conclusion, we found that oophorectomy is associated with a reduced risk of death from breast cancer in *BRCA2* carriers with breast cancer and should be considered in the treatment plan. Oophorectomy is also of value in preventing the second primary ovarian cancer. Further studies on the benefits of oophorectomy and of other endocrine therapies in this group of women are warranted.

## Supplementary information

Supplementary Tables

## Data Availability

All data relevant to the study are included in the paper.
