# Supplementary material for: Survival from breast cancer in women with a BRCA2 mutation by treatment
Source: Br J Cancer. 2021 Feb 18;124(9):1524–32. doi: 10.1038/s41416-020-01164-1 (PMC8076275; doi:10.1038/s41416-020-01164-1)
Supplement: Supplementary file 1 — Supplementary Tables [file 41416_2020_1164_MOESM1_ESM.docx]

Supplementary Table 1. Characteristics of study subjects by source

| Variables | Mean or frequency  N=189 (UK) | Mean or frequency  N=289 (Canada) | Mean or frequency  N=85 (Australia) | Mean or frequency  N=62 (Poland) | Mean and frequency  N=39 (Italy) |
| --- | --- | --- | --- | --- | --- |
| Date of Birth | 1964 (1936-1989) | 1958 (1932-1979) | 1958 (1933-1980) | 1969 (1942-89) | 1971 (1947-87) |
| Age at diagnoses | 44.7(26.4-69.4) | 44.1(27.0-69.0) | 45.2(27-70) | 45.3(26-78) | 44.1(23.0-67.4) |
| Age at gene testing  Before diagnosis  0-1 year after diagnosis  1-5 year after diagnosis  5+ years after diagnosis  Mean time elapsed from diagnosis to genetic testing (years) | 46.1(27.7-70.4)  20(10.6%)  49(25.9%)  120(43.5%)  0  1.7(0.0-5.0) | 44.3(26.4-70.0)  84(29.1%)  95(32.9%)  110(38.1%)  0  1.5(0-5) | 45.6(27.0-70.0)  16(18.8%)  57(67.1%)  12(14.1%)  0  0.7(0-2) | 47.2(26.4-73.5)  5(8.1%)  22(35.5%)  35(56.5%)  0  1.6(0.0-4.9) | 45.3(30.3-72.0)  5(12.8%)  19(48.7%)  15(38.5%)  0  1.3(0-4.6) |
| Age at last follow-up  Years of follow-up | 50.1(28.9-74.8)  5.4(0-24.8) | 53.4(31.8-80.0)  9.3(0.1-27.9) | 56.6(33.4-80.0)  10.9(1-20) | 49.2(30.0-77.2)  3.6(0.2-15.5) | 47.9(31.7-72.0)  3.8(0.2-12.1) |
| Contralateral breast cancer  Ovarian cancer | 8(4.2%)  5(2.7%) | 15(5.2%)  3(1.0%) | Not available  1(1.2%) | 6(9.7%)  2(3.2%) | 0  0 |
| Death  No  Yes  Cause of death  Breast cancer  Other  unknown no BC/missing | 148(78.3)  41(21.6)  40  1  0 | 246(85.1)  43(14.9)  36  2  5 | 64(75.3)  21(24.7)  9  5  7 | 56(90.3%)  6(9.7%)  5  1  0 | 38(97.4%)  1(2.6%)  1  0  0 |
| Tumor grade  I  II  III  Missing | 7(3.7%)  58(30.7%)  97(51.3%)  27(14.3%) | 35(12.1%)  93(32.2%)  74(25.6%)  87(30.1%) | 5(5.9%)  34(40.0%)  43(50.6%)  3(3.5%) | 0  0  0  62(100%) | 3(7.7%)  13(33.3%)  22(56.4%)  1(2.0%) |
| Tumour size (mm)  <=10  11-20  21+  Missing | 22.6(2-120)  20(10.6%)  59(31.2%)  74(39.2%)  36(19.1%) | 22.7(0-300)  64(22.2%)  86(29.8%)  99(34.3%)  40(13.8%) | 22.2(2-95)  22(25.9%)  32(37.7%)  29(34.1%)  2(5.1%) | 19.3(0.5-65)  15(24.2%)  29(46.8%)  16(25.8%)  2(3.2%) | 20.7(3.0-60.0)  6(15.4%)  16(41.0%)  15(38.5%)  2(5.1%) |
| ER status  Positive  Negative  Missing | 123(65.1%)  31(16.4%)  35(18.5%) | 190(65.7%)  46(15.9%)  53(18.3%) | 65(76.5%)  10(11.8%)  10(11.8%) | 47(75.8%)  12(19.4%)  3(4.8%) | 30(76.9%)  8(20.5%)  1(2.6%) |
| Lymph node status  Negative  Positive  Missing | 88(46.6%)  70(37.1%)  31(16.4%) | 133(46.0%)  118(40.8%)  38(13.2%) | 42(49.3%)  40(47.1%)  3(3.5%) | 31(50.0%)  30(48.4%)  1(1.6%) | 16(41.0%)  20(51.3%)  3(3.5%) |
| Endocrine therapy  Neither  Tamoxifen alone  AI alone  Both  Missing | 41(21.7%)  115(60.9%)  9(4.8%)  1(0.5%)  23(12.2%) | 100(34.6%)  110(38.1%)  19(6.6%)  8(2.8%)  52(18.0% | 30(35.3%)  39(45.9%)  8(9.4%)  8(9.4%)  0 | 13(21.0%)  37(59.7%)  0  2(3.2%)  10(16.1%) | 14(35.9%)  7(18.0%)  13(33.3%)  4(10.3%)  1(2.6%) |
| Chemotherapy  No  Yes  Missing | 46(24.3%)  91(48.2%)  52(27.5%) | 72(24.9%)  201(69.6%)  16(5.5%) | 13(33.3%)  50(58.8%)  11(12.9%) | 10(16.1%)  51(82.3%)  1(1.6%) | 13(33.3%)  26(66.7%)  0 |
| Contralateral Mastectomy  No  Yes  Missing | 132(69.8%)  57(30.2%)  0 | 112(38.9%)  171(59.2%)  6(2.1%) | 28(32.9%)  57(67.1%)  0 | 46(74.2%)  10(16.1%)  6(9.7%) | 27(69.2%)  12(30.8%)  0 |
| Oophorectomy  No  Yes  Oophorectomy within one year of breast cancer  Oophorectomy after one year post breast cancer  Mean years after BC oophorectomy | 130(68.8%)  59(31.2%)  9(15.3%)  50(84.8%)  3.8 (1.0-9.0) | 92(31.8%)  197(68.2%)  45(22.8%)  152(77.2%)  3.5 (1-17) | 19(22.4%)  66(77.7%)  15(22.7%)  51(77.3%)  2.6 (1-12) | 25(40.3%)  37(59.7%)  11(29.7%)  26(70.38%)  2.0 (1.0-5.0) n=26 | 21(53.9%)  18(46.2%)  2(11.1%)  16(88.9%)  1.9 (1.0-5.0)n=16 |

Supplementary Table 2. Comparison of subjects with and without oophorectomy

| Variables | No oophorectomy  N = 287 | Oophorectomy  N = 377 | P-value* |
| --- | --- | --- | --- |
| Year of Birth (range) | 1963 (1932 - 1989) | 1960 (1933-1985) | 0.002 |
| Age at diagnosis (years, range) | 44.0(26.0-69.4) | 44.9(27.0-70.9) | 0.21 |
| Year of diagnosis | 2006.7(1900-2019) | 2004.9(1990-2018) | 0.0008 |
| Age at gene testing  Mean time elapsed from diagnosis to genetic testing (years) | 44.9(26.4-72.8)  1.5(0-5) | 45.7(26.5-73.4)  1.4(0-5) | 0.30 |
| Age at last follow-up (years, range)  Years of follow-up | 49.8(28.8-80.0)  5.7(0.0-24.3) | 53.9(31.8-80.0)  8.9(0.4-27.9) | <0.0001  <0.0001 |
| Contralateral breast cancer  No  Yes  Missing | 254(94.8)  14(5.2)  19 | 296(95.2)  15(4.8)  66 | 0.83 |
| Death  No  Yes  Cause of death  Breast cancer  Ovarian cancer  Other  unknown /missing | 215(74.9)  72(25.1)  59  1  4  8 | 337(84.9)  40(10.6)  32  0  4  4 | <0.0001 |
| Tumor grade  I  II  III  Missing | 20(9.7)  76(36.7)  111(53.6)  80 | 30(10.8)  122(44.0)  125(45.1)  100 | 0.17 |
| Tumour size (mm)  <=10  11-20  21+  Missing | 40(16.5)  95(39.3)  107(44.2)  45 | 87(25.6)  127(37.4)  126(37.1)  37 | 0.03 |
| ER status  Positive  Negative  Missing | 188(79.7)  48(20.3)  51 | 267(81.9)  59(18.1)  51 | 0.50 |
| Lymph node status  Negative  Positive  Missing | 126(51.4)  119(48/6)  42 | 184(53.6)  159(46.4)  34 | 0.60 |
| Endocrine therapy  Neither  Tamoxifen alone  AI alone  Both  Missing | 85(33.6)  140(56.9)  18(7.3)  3(1.2)  41 | 113(34.0)  168(50.6)  31(9.3)  20(6.0)  45 | 0.02 |
| Chemotherapy  No  Yes  Missing | 74(31.8)  159(68.2)  54 | 91(25.9)  260(34.1)  26 | 0.13 |
| Contralateral Mastectomy  No  Yes  Missing | 197(70.6)  82(29.4)  8 | 148(39.7)  225(73.3)  4 | <0.0001 |

* missing data excluded in the test

Supplementary Table 3. Causes of death by oophorectomy status

| Cause of death | No Oophorectomy | Had oophorectomy |
| --- | --- | --- |
| Total deaths | 72 | 40 |
| Breast cancer | 59 (81.9%) | 32 (80.0%) |
| Ovarian cancer | 1 (1.4%) | 0 |
| Other cancer | 2 (2.8%) | 4 (10.0%) |
| Other | 2 (2.8%) | 0 |
| Missing | 8 (11.1%) | 4 (10.0%) |

Supplementary Table 4. Hazard ratios for death from breast cancer for selected variables. ER+ subjects only

| Variables | Cases/total | Univariate  HR(95%CI)P | Multivariate *  HR(95%CI)P |
| --- | --- | --- | --- |
| Age at dx  <=40  41-50  50+ | 27/166  21/174  12/115 | 1  0.76(0.43-1.35)0.36  0.72(0.36-1.42)0.34 | 1  0.98(0.54-1.78)0.95  0.70(0.34-1.43)0.32 |
| BC size (mm)  <=10  11-20  >20 | 3/82  25/170  27/172 | 1  4.97(1.50-16.5)0.009  6.00(1.82-19.8)0.003 | 1  3.40(1.00-11.5)0.05  4.16(1.20-14.5)0.02 |
| Endocrine therapy  None  Tamoxifen alone  AI alone  Both  Either | 9/75  41/269  3/43  1/18  45/330  36/330 (10 years) | 1  1.62(0.79-3.34)0.19  0.86(0.23-3.19)0.82  0.46(0.06-3.64)0.46  1.45(0.71-2.98)0.31 | 1  1.57(0.72-3.41)0.25  1.23(0.30-5.04)0.77  0.60(0.07-4.90)0.63  1.48(0.69-3.20)0.31 |
| BC grade  1 or 2  3 | 27/205  21/152 | 0.82(0.46-1.45)0.49  1 | 1.02(0.57-1.84)0.94  1 |
| Node status  Negative  Positive | 25/217  34/216 | 1  1.64(0.98-2.75)0.06 | 1  1.34(0.74-2.44)0.34 |
| Chemotherapy  N  Y | 12/124  37/288 | 1  1.49(0.78-2.87)0.23 | 1  1.05(0.49-2.24)0.90 |
| Contralateral mastectomy  Yes (time dependent)  Missing | 37/231  22/217  1/7 | 1  0.55(0.32-0.93)0.03 | 1  0.69(0.39-1.24)0.22 |
| Oophorectomy  No  Yes  Yes, ooph at dx;  Yes, 1-2 yrs after dx;  Yes, 3+ yrs after dx;  Yes, age at ooph<50  Yes, age at ooph>50 | 34/188  26/267  7/62  13/136  6/69  20/177  6/90 | 1  0.41 (0.24-0.69) 0.0007  0.52 (0.23-1.17) 0.11  0.41 (0.12-0.78) 0.007  0.32 (0.13-0.78) 0.01  0.47 (0.27-0.82) 0.008  0.28 (0.12-0.68) 0.005 | 1  0.48 (0.25-0.86) 0.01  0.62 (0.25-1.55) 0.31  0.49 (0.24-1.01) 0.05  0.37 (0.15-0.93) 0.03  0.52(0.28-0.98) 0.04  0.38(0.14-1.00) 0.05 |

* adjusted by all the variables; the 7 subjects missing PM data were supposed as no PM in the estimation on the RR of factors other than PM.
